# Supplementary material for: Biocompatible Catanionic Vesicles from Arginine-Based Surfactants: A New Strategy to Tune the Antimicrobial Activity and Cytotoxicity of Vesicular Systems
Source: Pharmaceutics. 2020 Sep 9;12(9):857. doi: 10.3390/pharmaceutics12090857 (PMC7557587; doi:10.3390/pharmaceutics12090857)
Supplement: Supplementary file 1 [file pharmaceutics-12-00857-s001.pdf]

pharmaceutics
MDPI

Supplementary Materials: Biocompatible Catanionic Vesicles from Arginine-Based Surfactants: A New Strategy to Tune the Antimicrobial Activity and Cytotoxicity of Vesicular Systems

Aurora Pinazo[1], Ramon Pons[1], Ana Marques[2], Maribel Farfan. [2], Anderson Ramos da Silva[1] and Lourdes Perez[1*]

Table S1. Mean diameter, polydispersity index, z-potential values and visual appearance of the catanionic mixtures at day 20. Photographs corresponding to the C3L and C3S systems: left 2.8, center 5.5, right 8.2.

.	Formulation	Size (Dh nm)	PdI	Z potential (mV)	Visual aspect
1 mM	2.8 C3S	183	0.100	-25	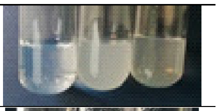
	5.5 C3S	515	0.230	+27	
	8.2 C3S	396	0.169	+39	
	2.8C3L		precipitate		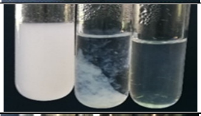
	5.5C3L		precipitate		
	8.2C3L	417	0.625	+46	
5 mM	2.8 C3S	227	0.226	-58	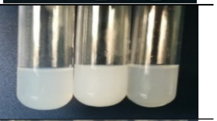
	5.5 C3S	487	0.261	+65	
	8.2 C3S	229	0.210	+66	
	2.8C3L		Gel		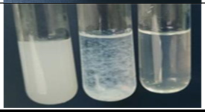
	5.5C3L		Precipitate		
	8.2C3L	388	0.623	viscous	

Table S2. Mean diameter, polydispersity index, z-potential values and visual appearance of the cholesterol containing catanionic mixtures (1 mM) at day 7 and 30. Photographs corresponding to the C3L and C3S systems: left 8.2, center 5.5 and right 2.8.

		DAY 7			DAY 30		
	Formulation	Size	PdI	Visual aspect	Size	PdI	Visual aspect
10% col	2.8C3L	683	0.373	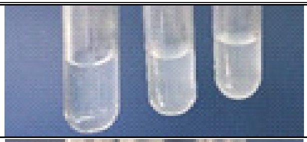	2745	1	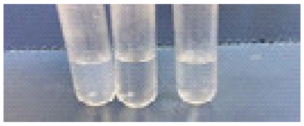
	5.5C3L	180	0.289		1458	0.485	
	8.2C3L	112	0.032		126	0.071	
	2.8 C3S	245	0.038	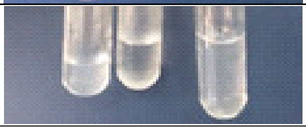	240	0.230	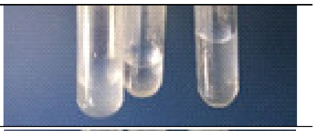
	5.5 C3S	683	0.512		563	0.613	
	8.2 C3S	173	0.125		182	0.119	
20% col	2.8C3L	926	0.532	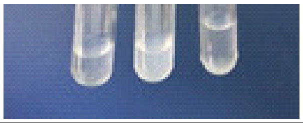	773	0.424	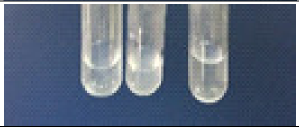
	5.5C3L	197	0.180		197	0.164	
	8.2C3L	164	0.342		161	0.223	
	2.8 C3S	255	0.372	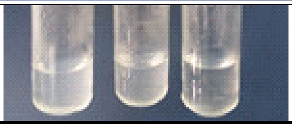	235	0.230	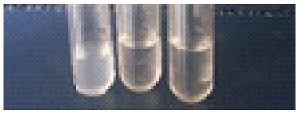
	5.5 C3S	412	0.344		244	0.351	
	8.2 C3S	196	0.191		171	0.399	

Table S3. MIC values (μM) of the pure C3(CA)2, 8-HS and sodium laurate.

	$C_3(CA)_2$	8-SHS	SL
S. aureus MRSA	9	>	>
B. subtilis	9	72	>
K. rhizophila	9	>	>
S. epidermidis	9	72	>
K. pneumoniae	>	>	>
P. aeruginosa	36	>	>
E. coli	36	>	>
C. albicans	18	>	>

Table S4. MIC values (μM) of catanionic mixtures.

	2.8.C3.S	5.5.C3.S	8.2.C3.S	2.8.C3.L	5.5.C3.L	8.2.C3.L
S. aureus MRSA	>	72	18	144	18	9
B. subtilis	>	72	36	72	18	9
K. rhizophila	>	72	18	144	18	4
S. epidermidis	>	36	18	144	18	9
K. pneumoniae	>	>	>	144	144	144
P. aeruginosa	>	144	72	-	72	72
E. coli	>	72	36	72	72	18
C. albicans	>	72	18	72	36	72

Table S5. MIC values (μM) of cholesterol containing (10%) catanionic mixture.

.	2.8.C3.S (10%)	5.5.C3.S (10%)	8.2.C3.S (10%)	2.8.C3.L (10%)	5.5.C3.L (10%)	8.2.C3.L (10%)
S. aureus MRSA	288	36	9	72	72	36
B. subtilis	288	36	72	288	36	36
K. rhizophila	288	36	9	72	72	36
S. epidermidis	288	36	9	288	36	9
K. pneumoniae	>	>	72	>	>	>
P. aeruginosa	>	36	36	>	72	36
E. coli	>	36	36	144	72	36
C. albicans	>	18	9	72	72	36

Table S6. MIC values (μM) of cholesterol containing (20%) catanionic mixtures.

	2.8.C3.S (20%)	5.5.C3.S (20%)	8.2.C3.S (20%)	2.8.C3.L (20%)	5.5.C3.L (20%)	8.2.C3.L (20%)
S. aureus MRSA	>	36	18	144	72	36
B. subtilis	>	72	72	288	288	36
K. rhizophila	>	36	18	>	72	36
S. epidermidis	288	36	18	288	288	9
K. pneumoniae	>	>	>	>	>	>
P. aeruginosa	>	72	36	>	>	72
E. coli	>	36	36	>	144	72
C. albicans	>	18	18	>	72	72

Table S7. MBC values (μM) of cholesterol containing (10%) catanionic mixtures.

	2.8.C3.S (10%)	5.5.C3.S (10%)	8.2.C3.S (10%)	2.8.C3.L (10%)	5.5.C3.L (10%)	8.2.C3.L (10%)
S. aureus MRSA	>	36	36	144	72	36
B. subtilis	288	72	288	288	144	72
K. rhizophila	288	36	9	72	72	144
S. epidermidis	>	36	9	288	36	18
K. pneumoniae	>	>	288	>	>	>
P. aeruginosa	>	36	36	>	144	36
E. coli	>	36	9	144	144	72
C. albicans	>	18	9	72	72	72

Table S8. MBC values (μM) of cholesterol containing (20%) catanionic mixtures.

	**2.8.C3.S (20%)**	**5.5.C3.S (20%)**	**8.2.C3.S (20%)**	**2.8.C3.L (20%)**	**5.5.C3.L (20%)**	**8.2.C3.L (20%)**
S. aureus MRSA	>	36	72	144	288	144
B. subtilis	>	72	144	288	>	36
K. rhizophila	>	36	18	>	72	288
S. epidermidis	>	36	36	288	288	36
K. pneumoniae	>	>	>	>	>	>
P. aeruginosa	>	288	36	>	>	72
E. coli	>	36	18	>	144	72
C. albicans	>	18	18	>	72	144

Table S9. Therapeutic index (HC_{50}/MIC) for the pure gemini surfactant and its catanaionic mixtures.

**.**	**$C_3(CA)_2$**	**2.8.C3.S**	**5.5.C3.S**	**8.2.C3.S**	**2.8.C3.L**	**5.5.C3.L**	**8.2.C3.L**
S. aureus MRSA	6.7	<1	2.2	4.4	>1	>11	12
B. subtilis	6.7	<1	2.2	2.2	>2.8	>11	12
K. rhizophila	6.7	<1	2.2	4.4	>1	>11	24
S. epidermidis	6.7	<1	4.4	4.4	>1	>11	12
K. pneumoniae	-	<1	<1	<1	>1	>1.3	0.8
P. aeruginosa	1.7	<1	1.2	1.1	-	>3	1.5
E. coli	1.7	<1	2.2	2.2	<2.8	>3	6
C. albicans	3.4	<1	2.2	4.4	<2.8	>3	1.5
		**2.8.C3.S (10%)**	**5.5.C3.S (10%)**	**8.2.C3.S (10%)**	**2.8.C3.L (10%)**	**5.5.C3.L (10%)**	**8.2.C3.L (10%)**
S. aureus MRSA		0.5	>4	9.4	>2	>2.2	2.8
B. subtilis		0.5	>4	1.2	-	>4.4	2.8
K. rhizophila		0.5	>4	9.4	>2	>2.2	2.8
S. epidermidis		0.5	>4	9.4	-	>4.4	5.6
K. pneumoniae		<0.5	-	1.2	-	-	<1
P. aeruginosa		<0.5	>4	2.5	-	>2.2	2.8
E. coli		<0.5	>4	2.5	>1	>2.2	2.8
C. albicans		<0.5	>8	9.4	>2	>2.2	2.8
		**2.8.C3.S (20%)**	**5.5.C3.S (20%)**	**8.2.C3.S (20%)**	**2.8.C3.L (20%)**	**5.5.C3.L (20%)**	**8.2.C3.L (20%)**
S. aureus MRSA		-	>4.4	>8.8	1	>2.2	>4.4
B. subtilis		-	>2.2	>2.2	-	-	>4.4
K. rhizophila		-	>4.4	>8.8	-	>2.2	>4.4
S. epidermidis		-	>4.4	>8.8	-	-	>16
K. pneumoniae		-	-	-	-	-	-
P. aeruginosa		-	>2.2	>4.4	-	-	>2.2
E. coli		-	>4.4	>4.4	-	>1	>2.2
C. albicans		-	>8.8	>8.8	-	>2.2	>2.2

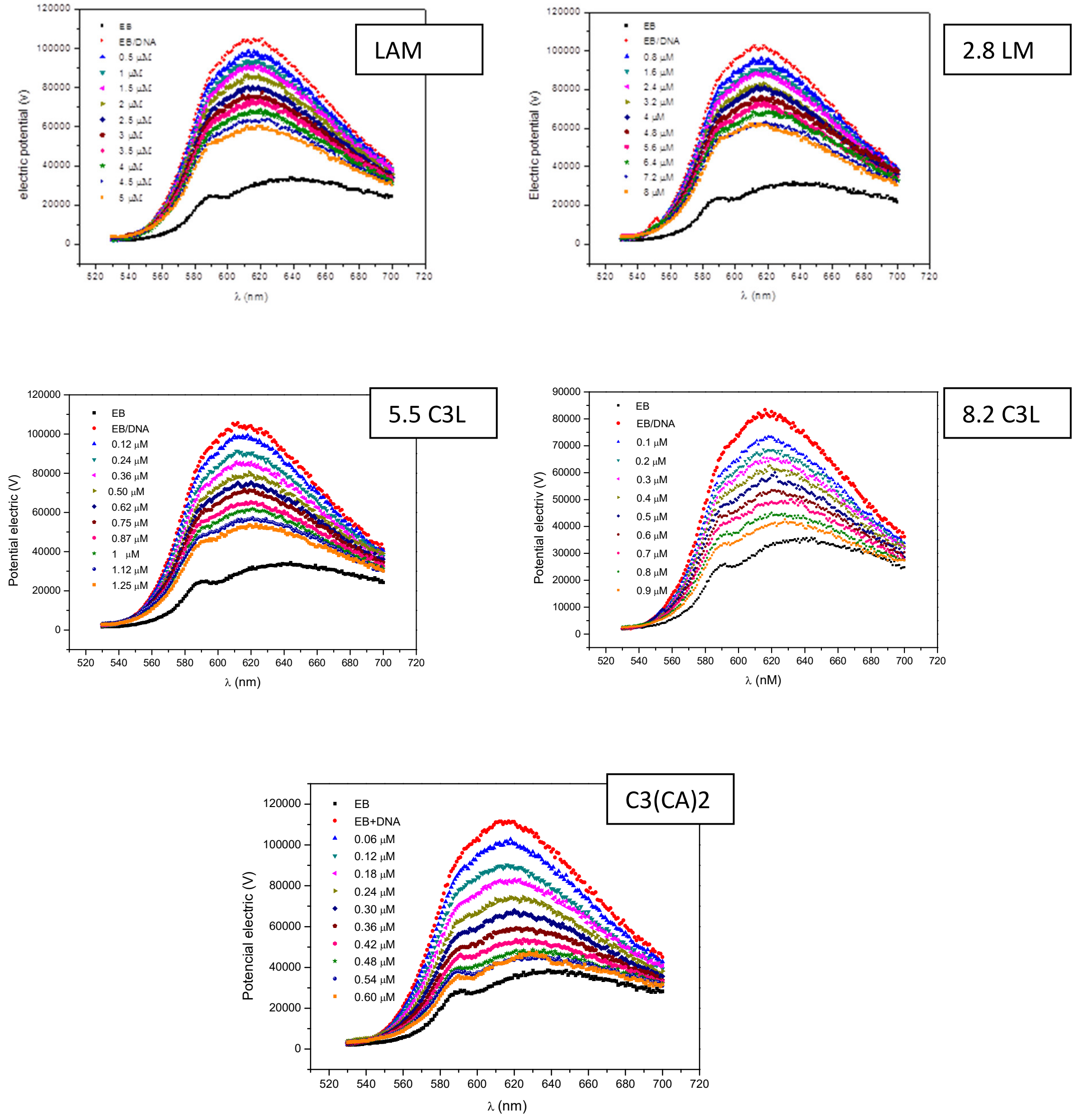

Figure S1. Ethidium bromide exclusion experiments for LAM, C3(CA)2 and their catanionic mixtures.
